# Pharmacogenomics of Rivaroxaban: Association of *CYP3A4, CYP3A5, CYP2J2, ABCB1*, and *ABCG2* Variants with Bleeding and Thrombotic Outcomes in Real-World Clinical Practice †

**DOI:** 10.3390/pharmaceutics18070884

**Published:** 2026-07-20

**Authors:** Ana Marija Slišković, Vladimir Trkulja, Lana Ganoci, Tamara Božina, Vedran Pašara, Majda Vrkić Kirhmajer, Jozefina Palić, Dominik Strikić, Marino Narančić, Ivana Sopek Merkaš, Iveta Merćep, Joško Bulum, Livija Šimičević

**Affiliations:** 1 Department of Cardiovascular Diseases, University Hospital Centre Zagreb, 10000 Zagreb, Croatia; vedran.pasara@gmail.com (V.P.); majda_vrkic@yahoo.com (M.V.K.); jbulum@gmail.com (J.B.); 2 Department of Basic and Clinical Pharmacology, University of Zagreb School of Medicine, 10000 Zagreb, Croatia; vladimir.trkulja@mef.hr (V.T.);; 3 Division of Pharmacogenomics and Therapy Individualization, Department of Laboratory Diagnostics, University Hospital Centre Zagreb, 10000 Zagreb, Croatia; jozefina.palic@outlook.com (J.P.); livija73@gmail.com (L.Š.); 4 Department of Medical Chemistry, Biochemistry and Clinical Chemistry, University of Zagreb School of Medicine, 10000 Zagreb, Croatia; tamara.bozina@mef.hr; 5 Department of Internal Medicine, University of Zagreb School of Medicine, 10000 Zagreb, Croatia; imercep@gmail.com; 6 Division of Clinical Pharmacology, Department of Internal Medicine, University Hospital Centre Zagreb, 10000 Zagreb, Croatia; strikic.dominik@gmail.com; 7 Division of Hematology, Department of Internal Medicine, University Hospital Centre Zagreb, 10000 Zagreb, Croatia; marinonarancic@gmail.com; 8 Department of Cardiology, Special Hospital for Medical Rehabilitation Krapinske Toplice, 49217 Krapinske Toplice, Croatia; ivana.sopek92@gmail.com

**Keywords:** rivaroxaban, pharmacogenomics, bleeding, direct oral anticoagulants, personalized medicine

## Abstract

Aim: To evaluate associations between polymorphisms in *CYP3A4* (*1B, *22), *CYP3A5* (*3), *CYP2J2* (*7, *rs11572325*), *ABCB1* (*c.1236C>T*, *c.2677G>T/A*, *c.3435C>T*, *rs4148738*) and *ABCG2* (*c.421C>A*) and the occurrence of bleeding or occlusive events in patients receiving rivaroxaban in real-world clinical practice. Methods: A nested case-control study, divided into two substudies (bleeding and thromboembolic events), was conducted within a prospective cohort of 385 adults receiving rivaroxaban at University Hospital Centre Zagreb (September 2021–September 2024). Bleeding events were classified per ISTH criteria, and genotyping was performed using TaqMan real-time PCR. Cases and controls were balanced using entropy balancing, and associations were estimated with Bayesian logistic regression under a skeptical prior N(0, 0.355); LASSO regression was used to identify clinical and genetic predictors of outcomes. Results: In total, 71 patients (18.4%) experienced bleeding events, most frequently gastrointestinal (47.9%), while 314 patients served as controls. No pharmacogenomic variant showed a clear association with bleeding risk (raw and balanced odds ratios 0.80–1.35; 95% credible intervals crossing 1.0). LASSO regression identified age (OR 2.00 per decade), gastrointestinal comorbidity (OR 8.77), and eGFR as the dominant predictors of bleeding. Twenty-one patients experienced occlusive events (15 venous, 6 arterial); however, the low event count precluded meaningful pharmacogenomic analysis. Conclusions: Individual pharmacogenomic variants in *CYP3A4*, *CYP3A5*, *CYP2J2*, *ABCB1*, and *ABCG2* together with pharmacogenetic-based phenotypes were not associated with clinically relevant bleeding in rivaroxaban-treated patients. Traditional clinical risk factors, particularly advanced age and gastrointestinal comorbidity, remain the dominant determinants of adverse outcomes. Routine pharmacogenomic testing to guide rivaroxaban dosing is not currently supported.

## 1. Introduction

Direct oral anticoagulants (DOACs)—factor IIa and factor Xa inhibitors—have fundamentally transformed the management of thromboembolic diseases over the past two decades. By offering predictable pharmacokinetics, fixed dosing regimens, and freedom from routine coagulation monitoring, DOACs have largely supplanted vitamin K antagonists (VKAs) for both stroke prevention in non-valvular atrial fibrillation (AF) and the prevention and treatment of venous thromboembolism (VTE) [1,2,3].

Rivaroxaban, the first of the direct Xa inhibitors, is metabolized primarily by hepatic cytochrome P450 enzymes CYP3A4 (~18%) and CYP2J2 (~14%), with additional contributions from CYP3A5, while its intestinal absorption and biliary/renal elimination are regulated by efflux transporters P-glycoprotein (P-gp, encoded by *ABCB1*) and breast cancer resistance protein (BCRP, encoded by *ABCG2*) [4,5].

The goal of pharmacogenetics is to identify gene variants, most commonly single nucleotide polymorphisms (SNPs), that underlie interindividual differences in drug response, and to implement pharmacogenetic testing in clinical practice in order to predict therapeutic response, reduce the risk of treatment inefficacy, and minimize the occurrence of adverse drug reactions (ADRs) [6,7].

Single nucleotide polymorphisms (SNPs) in these ADME (absorption, distribution, metabolism, excretion) genes can alter enzyme activity or transporter function, potentially modifying systemic drug exposure and thereby influencing clinical outcomes [8,9].

While the genotype represents an individual’s unique genetic constitution, the phenotype reflects their actual metabolic response. The phenotype results from interactions between the genotype and environmental factors (e.g., diet, smoking, co-medication), and therefore does not always fully correspond to the genotype [10].

Based on the genotype, five phenotypes are distinguished according to the rate and capacity of drug metabolism: poor, intermediate, normal, rapid and ultrarapid metabolizers (PM; IM; NM; RM; UM) [11].

Genome-wide association studies (GWAS) have led to the discovery of numerous variants associated with the development of adverse drug reactions, further increasing the interest of the pharmaceutical industry, regulatory bodies, and the academic community in the genetic basis of variable therapeutic response.

Despite a plausible biological rationale, evidence linking specific pharmacogenomic variants to clinical outcomes in rivaroxaban-treated patients remains heterogeneous and inconsistent [12,13,14,15,16,17].

A 2025 systematic review identified 25 polymorphic loci across *ABCB1*, *ABCG2*, *CYP3A4*, *CYP3A5*, *CYP2J2*, and other pharmacogenes associated with altered rivaroxaban pharmacokinetics, yet concluded that evidence is insufficient for routine clinical implementation due to significant inter-study heterogeneity [18]. Furthermore, pharmacokinetic signals do not necessarily translate into measurable clinical effects, particularly for drugs with a relatively wide therapeutic window such as rivaroxaban [19].

Although direct oral anticoagulants (DOACs), including rivaroxaban, demonstrate a more favorable pharmacokinetic profile relative to vitamin K antagonists (VKAs), considerable interindividual variability in plasma drug concentrations remains, the underlying determinants of which have not been fully elucidated. Given the established roles of cytochrome P450 enzymes and membrane transport proteins in the metabolism and elimination of rivaroxaban, it is postulated that genetic variants in *CYP3A4*, *CYP3A5*, *CYP2J2*, *ABCB1*, and *ABCG2* may contribute to this variability, as well as to the susceptibility to adverse drug reactions. The objective of the present study was to examine the association between the aforementioned pharmacogenetic variants and the occurrence of adverse drug reactions in patients receiving rivaroxaban therapy, with the aim of advancing the understanding of factors governing individual therapeutic response.

The present study aimed to assess the association of clinically relevant polymorphisms in *CYP3A4*, *CYP3A5*, *CYP2J2*, *ABCB1*, and *ABCG2* with bleeding and occlusive (thrombotic) events in a real-world cohort of patients receiving rivaroxaban according to approved indications at a tertiary cardiovascular center in Croatia.

To the best of our knowledge, the present nested case-control study constitutes one of the more comprehensive real-world assessments of multiple pharmacogenomic variants across the key CYP enzymes and ABC transporters implicated in rivaroxaban pharmacokinetics, evaluated against clinically defined bleeding and thromboembolic outcomes.

## 2. Materials and Methods

### 2.1. Study Outline

This nested case-control study was conducted at the Department of Cardiovascular Diseases and the Clinical Department of Laboratory Diagnostics, University Hospital Centre Zagreb (UHC Zagreb), Croatia, between September 2021 and September 2024, as part of the Croatian Science Foundation project PGxCardioDrug (UIP-2020-02-8189) [20]. The study was approved by the Ethics Committees of the University of Zagreb School of Medicine (380-59-10106-20-111/125) and UHC Zagreb (02/21 AG). The pharmacogenes of interest were *CYP3A4* *1B (rs2740574) and *22 (rs35599367); *CYP3A5* *3 (rs776746); *CYP2J2* *7 (rs890293) and rs11572325; *ABCB1* c.1236C>T (rs1128503), c.2677G>T/A (rs2032582), c.3435C>T (rs1045642), and rs4148738; and *ABCG2* c.421C>A (rs2231142). Combined CYP3A phenotype was assigned based on both CYP3A4*22 and CYP3A5*3 allelic status [21], and these phenotypes were further defined according to Clinical Pharmacogenetics Implementation Consortium (CPIC) guidelines [11].

**Figure 1:**
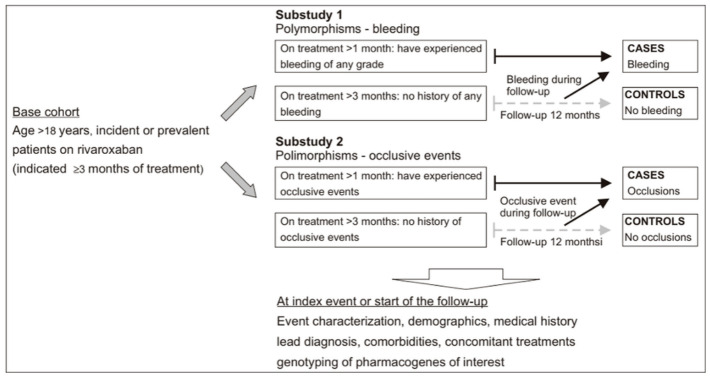
Study outline. Adults requiring ≥3 months of rivaroxaban treatment were followed up to occurrence of bleeding or occlusive events, and over a subsequent 12-month follow-up (a patient could have experienced more than one of either or both events). Data were used in two substudies based on the case-control principle: (i) Substudy 1 explored polymorphisms of interest with respect to bleeding events. Cases were patients treated for at least 1 month who developed bleeding. Controls were selected among patients with at least 3 months of a previous treatment without bleeding based on developments over a subsequent 12-month follow-up: if bleeding occurred, they were considered cases, if not, they were considered controls; (ii) Substudy 2 explored polymorphisms with respect to occlusive events. Cases and controls were identified on the same principles as in Substudy 1.

The base cohort comprised adult patients treated with rivaroxaban (incident, prevalent) for an indication requiring ≥3 months of treatment (Figure 1). Two substudies/analyses were performed on a case-control principle (Figure 1): (i) Substudy 1 explored association between polymorphisms and bleeding events. Cases were patients who experienced bleeding after at least 1 month of treatment had elapsed. Potential controls were patients who had been on treatment ≥3 months with no bleeding events over the previous period and over a 12-month follow-up. If, during the follow-up, they developed bleeding—they were counted as “cases”, if not, they were considered “controls” (Figure 1); (ii) Substudy 2 explored associations between polymorphisms and occlusive events: cases had experienced such events, controls were free of such events at the time of inclusion and over the subsequent 12-month follow-up.

### 2.2. Patients, Patient Management and Follow-Up

Eligible were adults with documented indication for rivaroxaban therapy of at least 3 months who provided written informed consent for genotyping of the pharmacogenes of interest, and for the use of anonymized data for research purposes and publication. Patients with significant thrombocytopenia (<100 × 10^9^/L), hepatic dysfunction (Child–Pugh B or C), severe renal impairment (eGFR < 15 mL/min/1.73 m^2^), and indications in which rivaroxaban is not routinely recommended (mechanical heart valves, rheumatic mitral stenosis, antiphospholipid syndrome) were not included.

For some of the “cases” (either bleeding or occlusive event), the time of enrollment was the time of the index event, whereas for others, as well as for the controls, time of enrollment was the start of the 12-month follow-up. For cases, data were collected at the index event/enrollment, or at the start of the follow-up and at a subsequent index event (and these were considered in the analysis). For controls, data were collected at the start of the follow-up. Collected were data on demographics, indication for and dose of rivaroxaban, comorbidities, concomitant treatments, and the estimate of the renal function: at the start of the rivaroxaban treatment (to guide dose selection), estimated glomerular filtration rate (eGFR) was recorded based on the CKD-EPI creatinine equation [22].

Patients also provided blood samples for genotyping the pharmacogenes of interest. Concomitant medication was evaluated for potential drug–drug interactions at the level of CYP enzymes and transporters (CYP3A4, CYP3A5, CYP2J2, ABCB1, ABCG2) and classified as inducers, inhibitors, substrates based on the LexiComp^®^ [23] and DrugBank databases [24].

Patients were managed as outpatients with visits scheduled in agreement with their main diagnosis and comorbidities, and no procedure beyond the standard of care procedures was undertaken for the purpose of this study. At each visit, detailed and targeted medical histories and—when indicated—clinical/laboratory investigations were taken in order to identify the outcomes of interest. Since practically all included patients originated from the Hospital’s administrative catchment population, the institutional electronic medical records were periodically searched for the same purpose. After the 12-month follow-up had elapsed, all patients were re-checked to confirm their case or control status, either in person or through a telephone contact.

All events were identified descriptively and characterized regarding severity. Bleeding events were classified per International Society on Thrombosis and Haemostasis (ISTH) criteria [25,26] as major (fatal, in critical organ, or causing Hb drop ≥ 20 g/L or requiring ≥2 units of packed red cells) or clinically relevant non-major (CRNM).

### 2.3. Genotyping

Genomic DNA was extracted from peripheral blood using the QIAamp DNA Mini Kit (Qiagen, Hilden, Germany). Genotyping of ten SNPs was performed using validated TaqMan SNP/DME Genotyping Assays on a Real-Time PCR System (Applied Biosystems, Foster City, CA, USA): *CYP3A4* *1B (rs2740574) and *22 (rs35599367); *CYP3A5* *3 (rs776746); *CYP2J2* *7 (rs890293) and rs11572325; *ABCB1* c.1236C>T (rs1128503), c.2677G>T/A (rs2032582), c.3435C>T (rs1045642), and rs4148738; *ABCG2* c.421C>A (rs2231142).

### 2.4. Statistical Analysis

The main analysis intended to assess associations between individual polymorphisms or genotype-predicted phenotypes and the case status (separately in each substudy): in a series of consecutive analyses (one per polymorphism/phenotype), cases and controls were balanced on a range of covariates except for the polymorphisms/phenotype of interest using entropy balancing, and then (weighted) logistic regression models were fitted to the case status. Entropy balancing balances distributional moments (mean, variance, skewness, kurtosis) between cases and controls without excluding participants. It was performed using package WeightIt [27] in R language and environment for statistical computing (Version 4.5.0) (R Foundation for Statistical Computing). Bayesian logistic models [package rstanarm in R [28]] were fitted to the data with a moderately informed skeptical prior on ln(OR) [N(0, 0.355)] which assigns 95% probability to odds ratios (ORs) between 0.50 and 2.00, in line with the general views on contributions of individual pharmacogenetic polymorphisms to clinical outcomes, and in line with predominantly null findings in previous research. Raw and adjusted (weighted) ORs were generated (95% credible intervals). As a supplementary analysis, all collected clinical and genetic variables were considered as potential predictors of the case status. Least absolute shrinkage and selection operator (LASSO) logistic regression models were used (proc glmselect in SAS 9.4 for Windows, SAS Inc., Cary, NC, USA) to identify variables associated with the outcomes (Akaike’s information criterion). Hardy–Weinberg equilibrium was verified for all loci prior to the analysis [package genetics in R [28]].

## 3. Results

### 3.1. Cohort Characteristics

A total of 385 patients (median age 67 years, IQR 58–74); 238 (61.8%) males and 147 (38.2%) females were enrolled. Some of them suffered from several conditions that required rivaroxaban treatment, the most common being nonvalvular atrial fibrillation (AF)/atrial flutter (AFL) (284, 73.8%), followed by chronic coronary syndrome (95, 24.7%), peripheral arterial disease (68, 17.7%), ischaemic stroke/transient ischaemic attack (TIA) (57, 14.8%), and VTE (52, 13.5%). The most common rivaroxaban dose was 20 mg once daily (273, 70.9%). Median eGFR was 75 mL/min/1.73 m^2^ (IQR 58–89). The most frequent comorbidities were hypertension (82.3%), dyslipidaemia (69.6%), and diabetes mellitus (22.1%).

The majority of participants carried the *CYP3A4* wild-type genotype (*1/*1: 96.4% for *1B, 93.8% for *22). CYP3A5*3/*3 (non-expressor) genotype predominated (89.6%) (Table 1). The combined CYP3A phenotype was intermediate metabolizer (IM) in 83.6%, rapid metabolizer (RM) in 10.1%, and poor metabolizer (PM) in 6.2% (Table 1). CYP2J2*7 was observed in 11.9%. For *ABCG2* c.421C>A and *CYP2J2* c.A>T (rs11572325), 18.5% and 21.5%, respectively, carried at least one variant allele. The *ABCB1* variant allele frequencies were substantially higher (40.1–53.9%) (Table 1).

### 3.2. Bleeding Events

**Figure 2:**
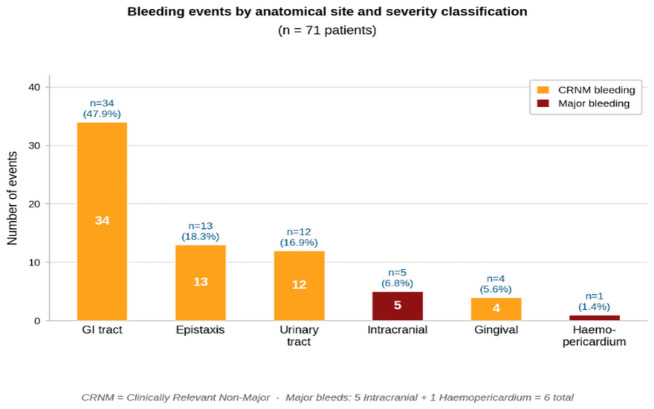
Bleeding events by anatomical site and severity (n  =  71 patients, 74 events). Bars show clinically relevant non-major (CRNM) (orange) and major (dark red) bleeding by site; event counts (n) and site percentages annotated above each bar. GI—gastrointestinal.

Seventy-one patients (18.4%) experienced at least one bleeding event (cases), with 74 total episodes (three patients had two concurrent manifestations). Six major bleeds were recorded (5 intracranial hemorrhages; 1 hemopericardium), whereas the most frequent non-major bleeding sites were gastrointestinal (34, 47.9%) (Figure 2).

Duration of rivaroxaban treatment was longer in controls than in cases (median 24 months, range 15–108 vs. median 15 months, range 3–96) (see also Supplementary Material Figure S1). Cases and controls somewhat differed in a range of demographic and comorbidity characteristics (Table 2), but only a few differences appeared marked: cases were older (74 vs. 66 years) and had lower eGFR (69 vs. 76 mL/min/1.73 m^2^) and higher prevalence of gastrointestinal comorbidities (45.1 vs. 7.6%) and malignancy (28.1 vs. 16.3%), more commonly used the 15 mg/day dose (28.2 vs. 10.5%), and less commonly used the 20 mg/day dose (57.8 vs. 73.2%) (Table 2).

The use of relevant comedication appeared similar in cases and controls (Table 3).

Also, prevalence of the investigated polymorphisms and genotype-predicted phenotypes appeared similar in cases and controls (Table 4).

Since the number of patients with a poor metabolizer CYP3A phenotype and with reduced-function phenotype based on CYP2J*7 was low, relationship between these polymorphisms and bleeding could not be meaningfully evaluated. For the remaining six polymorphisms, dichotomized to variant allele carriage and wild type genotype, entropy balancing enabled perfect balance between cases and controls on a number of covariates (Supplementary Material Tables S1–S6). Differences in age, eGFR and prevalence of gastrointestinal comorbidities were such that balance could not be achieved; hence, these three variables were used as covariates in the analysis of raw and balanced (weighted) data: neither raw nor the fully adjusted analyses indicated association of any of these polymorphisms with the risk of bleeding (Table 5).

**Figure 3:**
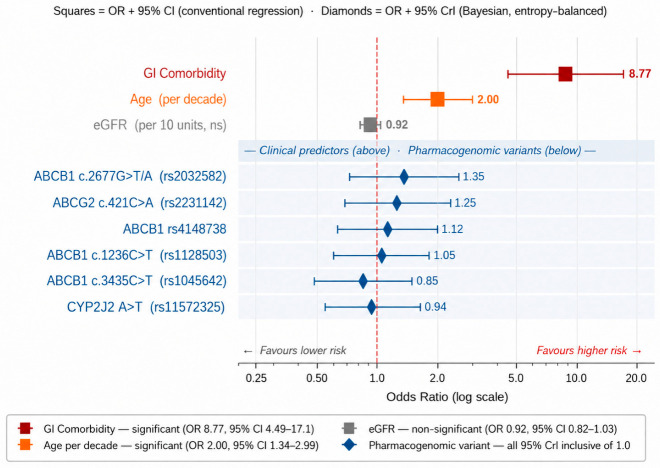
Comparative effect sizes of clinical predictors versus pharmacogenomic variants for bleeding outcome (log-scale). Squares (clinical factors) versus diamonds (pharmacogenomic variants). GI comorbidity (OR 8.77) and age per decade (OR 2.00) dominate outcome prediction. All pharmacogenomic variants cluster tightly around the null (OR 0.85–1.35) with overlapping credible intervals. GI—gastrointestinal.

In the supplementary analysis, among the range of demographic, clinical and genetic variables, the LASSO regression selected only three variables associated with the outcome: age, where older age was associated with a higher odds of bleeding (OR 2.00, 95%CI 1.34–2.99 per 10 years); gastrointestinal comorbidity, strongly associated with the risk of bleeding (OR = 8.77, 95%CI 4.49–17.1); and eGFR, where higher eGFR tended to be associated with a lower risk of bleeding. Figure 3 illustrates the discrepancy between the strong associations of age and gastrointestinal comorbidity with the risk of bleeding, and the lack of association between the investigated polymorphisms and the outcome.

### 3.3. Occlusive Events

A total of 21 patients experienced occlusive events: 15 venous thromboembolic events (10 DVT, 5 PE) and 6 arterial occlusions (3 ischemic strokes, 2 TIA, 1 peripheral arterial occlusion). The number of cases was too low for a meaningful analysis of associations between the polymorphisms of interest and this outcome.

## 4. Discussion

In this real-world study, no clear or consistent association was observed between the investigated pharmacogenetic variants and bleeding or thromboembolic outcomes in rivaroxaban-treated patients. In contrast, advanced age, gastrointestinal comorbidity, and renal function emerged as the main predictors of bleeding risk, suggesting that clinical factors may be more important determinants of adverse outcomes than individual genetic variants. Prevalence of genotypes and genotype-predicted phenotypes in this study cohort corresponds to those reported for European reference populations, including the marked predominance of CYP3A5 non-expressors (*3/*3; 89.6%) [29,30], low variant allele frequency of CYP2J2*7 (6.0%) concordant with 5.49% observed in Caucasians [31].

Prevalence of *ABCG2* c.421 C>A (9.6%) correlates with previous study of this polymorphism in the Croatian population [32] and aligns with European reference data (∼5–10%) [33] together with the common *ABCB1* variants (40.1–53.9%) [34]. The distribution of the analyzed polymorphisms was consistent with European populations.

Due to the marked predominance of the CYP3A rapid/intermediate metabolizer phenotype and the CYP2J2 normal phenotype according to the *7 allele among cases and controls, it was not possible to reliably assess their association with bleeding risk, and for other evaluated variants neither raw nor the fully adjusted analyses indicated association of any of these polymorphisms with the risk of bleeding reflecting that no polymorphism showed a significant independent association with the outcomes.

Our findings thus suggest that, in patients treated with rivaroxaban at standard guideline-recommended dosing, the risk of adverse events is determined primarily by clinical predictors, whereas the contribution of individual pharmacogenetic variants is likely limited.

Results of this study showed that age, gastrointestinal comorbidity, and eGFR were the only variables identified by the LASSO model as relevant predictors, with older age and gastrointestinal comorbidity being clearly associated with a higher risk of adverse events during rivaroxaban treatment.

In Substudy 1 (Bleeding) cases were older (median 74 vs. 66 years) and had a substantially higher prevalence of gastrointestinal comorbidities (45.1% vs. 7.6%) and malignancy (28.1% vs. 16.3%). The 15 mg/day dose was more frequent among cases (28.2% vs. 10.5%), reflecting confounding by indication. Pharmacogenomic variant frequencies did not differ materially between cases and controls. This finding is expected and consistent with known biological mechanisms. Older age is associated with multiple comorbidities, including the increasing prevalence of cancer in the elderly, alterations in drug pharmacokinetics and pharmacodynamics, increased propensity for bleeding [35,36], whereas gastrointestinal disorders provide a direct anatomical and pathophysiological substrate for bleeding [37].

In Substudy 2 (Occlusive Events), the low event count, just 21 thromboembolic cases, precluded reliable pharmacogenomic analysis. In addition, no pharmacogenomic variant showed a clear signal (all OR 0.93–1.19; 95% CrIs inclusive of 1.0). Nevertheless, the low number of thromboembolic events—only 21 in the cohort (10 DVT, 5 PE, and 6 arterial occlusions: 3 ischemic strokes, 2 TIAs, and 1 peripheral arterial occlusion) among 385 patients—suggests the efficacy and effectiveness of rivaroxaban for its most common indication, namely the prevention of venous and systemic thromboembolism, even at the cost of six major bleeding events (five ICH, one hemopericardium).

Existing studies evaluating the pharmacogenetics of rivaroxaban have produced inconsistent findings. In the present study, neither *CYP3A4* nor *CYP2J2* variants were associated with clinical outcomes.

Although CYP3A4*22 variant has well-established functional consequences for several drugs, including tacrolimus and statins [38], its impact on rivaroxaban remains unclear. Previous studies have reported conflicting results: Li et al. identified associations between *CYP3A4* variants and minor bleeding in an AF cohort [13], whereas Wu et al. [12] found no such effect. Likewise, CYP2J2*7 was not associated with clinical outcomes in our cohort, despite the recognized contribution to rivaroxaban metabolism [4]. CYP2J2 additionally metabolizes arachidonic acid to cardioprotective epoxyeicosatrienoic acids, introducing potential pleiotropy that may complicate genotype–outcome relationships [39].

Studies investigating *ABCB1* polymorphisms in DOAC-treated patients have yielded inconsistent results [15,18,40,41,42]. Wang et al. identified an association between the *ABCB1* c.3435T/T with increased risk of VTE [15], while a Korean study reported an association between *ABCB1* rs1045642 and bleeding events [14]. In contrast, neither individual *ABCB1* variants nor dominant haplotype were associated with bleeding or thromboembolic outcomes in our cohort. The two most prevalent haplotypes both predicted net-normal transporter function, reducing the likelihood of detecting a haplotype-mediated pharmacokinetic effect.

The *ABCG2 c.421C>A* variant is known to reduce BCRP-mediated efflux and is associated with higher rivaroxaban exposure, with higher AUC and Cmax values in pharmacokinetic studies [43,44], yet consistent translation to clinical bleeding risk has not been demonstrated [12,18,45], which is in line with the findings of the present study.

Our findings are broadly consistent with several recent studies. The retrospective cohort by Campos-Staffico et al., comprising 2364 patients and evaluating eight pharmacokinetic variants across *ABCB1*, *ABCG2*, *CYP2J2*, and *CYP3A4/5*, likewise found no significant association between pharmacogenomic profile and DOAC-related bleeding [19], while the prospective multicenter analysis of Wu et al. reached the same conclusion for the same gene panel [12]. Furthermore, the recent systematic review concluded that the current evidence remains insufficient to support routine pharmacogenomic-guided rivaroxaban dosing [18], an interpretation consistent with our nested case-control cohort. The inconsistencies among published studies likely reflect heterogeneity in study design, outcome definition, ethnic allele-frequency distribution, and sample size, and highlight the difficulty of detecting modest pharmacogenomic effects in the presence of dominant clinical determinants. In our cohort, advanced age and gastrointestinal comorbidity emerged as the principal predictors of bleeding, reinforcing the view that clinical risk stratification, not genotype, currently provides the more actionable basis for individualizing rivaroxaban therapy. Comparison of key pharmacogenomic studies evaluating rivaroxaban clinical outcomes is shown in Table 6.

The absence of a detectable pharmacogenomic effect may be explained by several biological and methodological factors. First, rivaroxaban undergoes metabolism via several parallel pathways, so, reduced activity of a single enzyme or transporter may be compensated by alternative routes, attenuating the net pharmacokinetic impact of any single genetic variant [19]. Second, unlike warfarin, whose narrow therapeutic index renders pharmacogenomic variability clinically decisive, rivaroxaban has a relatively broad therapeutic window, meaning that modest concentration changes driven by individual SNPs are unlikely to generate a clinically relevant association [19]. Third, the number of participants carrying rare genotypes was small, particularly for *CYP3A4* and *CYP2J2* variants, limiting the statistical power to detect associations.

Our study has several limitations. First, the research was conducted at a single center. Although the study had sufficient statistical power for the primary endpoint, bleeding (71 cases), the number of occlusive events (N = 21) was too small to allow reliable assessment of pharmacogenetic associations. Consequently, substantially larger multicenter studies will be required to adequately evaluate pharmacogenomic determinants of occlusive outcomes.

Additionally, plasma rivaroxaban concentrations and anti-Xa activity were not measured, precluding direct assessment of genotype–pharmacokinetics relationships, while treatment adherence was not directly quantified. Measurement of plasma rivaroxaban concentrations was not included in the original study protocol, which was designed to evaluate pharmacogenetic associations with clinical outcomes rather than pharmacokinetic parameters. Furthermore, accurate assessment of rivaroxaban plasma concentrations requires strictly time-controlled blood sampling (e.g., at peak [Cmax] or trough [Ctrough] concentrations), which is logistically challenging in routine clinical practice.

Consequently, the lack of pharmacokinetic data limits mechanistic interpretation of the observed genotype–outcome relationships. Previous pharmacokinetic studies have demonstrated that certain variants, particularly *ABCG2 c.421C>A*, are associated with increased rivaroxaban exposure [43,44], whereas consistent associations between these pharmacokinetic differences and bleeding or thrombotic outcomes have not been demonstrated [18]. Similarly, recent studies evaluating *CYP3A4/5*, *ABCB1*, *ABCG2* and *CYP2J2* variants have reported modest effects on rivaroxaban pharmacokinetics but absent effects on clinical outcomes, particularly bleeding risk [12]. In line with these observations, Nakagawa et al. concluded that the dose-adjusted plasma trough concentration ratio (C_0h_/D) of rivaroxaban did not differ significantly among *ABCB1* c.3435C>T, *c. 2677 G>A/T*, *c.1236 C>T*, *ABCG2 c.421C>A*, CYP3A5*3 and CYP2J2*7 genotypes [17].

However, to our knowledge, this nested case–control study represents one of the larger real-world evaluations of multiple pharmacogenomic variants in the principal CYP enzymes and ABC transporters involved in rivaroxaban disposition, assessed against clinically defined hemorrhagic and thrombotic outcomes. Despite a biologically plausible rationale and the application of advanced statistical methods, no clear or consistent pharmacogenomic signal was identified. Clinical factors, particularly advanced age (OR 2.00 per decade) and gastrointestinal comorbidity (OR 8.77 for bleeding), far outweighed any genetic contribution in determining outcome risk.

Our results align with extensive real-world evidence showing that rivaroxaban’s GI bleeding risk is driven primarily by patient characteristics [37], thereby supporting current guideline recommendations. On the other hand, the recent COBRRA randomized trial demonstrated that the choice of DOAC itself is a powerful modifiable determinant of bleeding: among patients with acute VTE, apixaban approximately halved clinically relevant bleeding compared with rivaroxaban (3.3% vs. 7.1%; RR 0.46, 95% CI 0.33–0.65) without compromising efficacy [46].

Additional interesting findings come from network meta-analysis comparing real-world effectiveness and safety of all DOACs (apixaban, dabigatran, edoxaban, rivaroxaban) vs. VKAs in European NVAF patients. DOACs showed benefit over VKAs for most outcomes, of which major bleeding and all-cause mortality were most commonly reported. Edoxaban demonstrated a comparable effectiveness/safety profile to other DOACs and significantly reduced risk of major bleeding (hazard ratio [95% credible interval]: 0.67 [0.54, 0.84]) and intracranial hemorrhage (0.69 [0.51, 0.94]) versus rivaroxaban [47]. In light of these findings, apixaban is associated with a more favorable safety profile than rivaroxaban in patients with VTE, whereas edoxaban demonstrates a more favorable safety profile than rivaroxaban in patients with AF, as both DOACs are associated with a lower risk of bleeding.

Validated clinical risk scores, such as HAS-BLED [48] and ORBIT [49], which incorporate age, renal function, and GI disease status should guide rivaroxaban prescribing and monitoring, independent of pharmacogenomic testing. In the present study, variant carrier status was not associated with bleeding or occlusive events. This discrepancy between pharmacogenetic and clinical findings highlights that drug exposure alone does not determine outcome risk within standard dosing regimens. Consequently, our results do not support routine testing of the analyzed variants to guide rivaroxaban dosing or patient selection in current clinical practice.

Nevertheless, pharmacogenetic testing may still prove helpful in selected, high-risk situations (e.g., with unexpectedly severe bleeding, atypical therapeutic response or complex polypharmacy). However, further large, prospective studies integrating pharmacokinetic biomarkers, polygenic approaches, and drug–drug–gene interactions are needed before implementation in routine clinical practice can be considered.

## 5. Conclusions

In conclusion, our results suggest that routine testing of the analyzed variants is unlikely to improve risk stratification in rivaroxaban-treated patients. In contrast, traditional clinical risk factors (older age and gastrointestinal comorbidity) remain the dominant determinants of adverse outcomes, primarily bleeding risk. None of the investigated polymorphisms showed a significant independent association with clinical outcomes.

Future research should integrate pharmacokinetic biomarkers with polygenic risk approaches and drug–drug–gene interaction analyses in larger, multicenter prospective cohorts to improve predictive performance compared with the single-variant approach used in the present study.

## Figures and Tables

**Table 1:** Prevalence of genotypes and genotype-predicted phenotypes in the study cohort (N = 385).

CYP Enzymes	n (%)	Transporters	n (%)
CYP3A4*1B (rs2740574) genotype		*ABCG2* c.421C>A (rs2231142)	
*1/*1	371 (96.4)	C/C	314 (81.5)
*1/*3	13 (3.4)	C/A	68 (17.7)
*3/*3	1 (0.2)	A/A	3 (0.8)
Variant allele frequency	1.9%	Variant allele frequency	9.6%
CYP3A4*22 (rs35599367) genotype		ABCG2 phenotype per rs2231142	
*1/*1	361 (93.8)	Normal activity	314 (81.5)
*1/*22	23 (6.0)	Reduced activity	71 (18.5)
*22/*22	1 (0.2)	*ABCB1* c.1236C>T (rs1128503)	
Variant allele frequency	3.2%	C/C	124 (32.2)
CYP3A4 phenotype		C/T	183 (47.5)
Normal metabolizer (NM)	361 (93.8)	T/T	78 (20.3)
Intermediate metabolizer (IM)	23 (6.0)	Variant allele frequency	44.0%
Poor metabolizer (PM)	1 (0.2)	*ABCB1* c.2677G>T/A (rs2032582)	
CYP3A5*3 (rs776746) genotype		G/G	158 (41.0)
*1/*1	2 (0.5)	G/T or G/A	145 (37.7)
*1/*3	38 (9.9)	T/T or T/A or A/A	82 (21.3)
*3/*3	345 (89.6)	Variant allele frequency	40.1%
Variant allele frequency	94.5%	*ABCB1* c.3435C>T (rs1045642)	
CYP3A5*3 phenotype		C/C	89 (23.1)
Enzyme expressors	40 (10.4)	C/T	177 (46.0)
Non-expressors	345 (89.6)	T/T	119 (30.9)
CYP3A combined phenotype		Variant allele frequency	53.9%
Poor metabolizer (PM)	24 (6.2)	ABCB1 phenotype per rs1045642	
Intermediate metabolizer (IM)	322 (83.6)	Normal function	266 (69.1)
Rapid metabolizer (RM)	39 (10.1)	Reduced function	119 (30.9)
CYP2J2*7 (rs890293) genotype		*ABCB1* c.2482-2236G>A (rs4148738)	
*1/*1	339 (88.1)	G/G	109 (28.3)
*1/*7	46 (11.9)	G/A	184 (47.8)
*7/*7	0	A/A	92 (23.9)
Variant allele frequency	6.0%	Variant allele frequency	47.8%
CYP2J2*7 phenotype		ABCB1 phenotype per rs4148738	
Normal activity	339 (88.1)	Normal activity	109 (28.3)
Reduced activity	46 (11.9)	Increased/extensive activity	276 (71.7)
*CYP2J2* c.A>T (rs11572325)			
A/A	302 (78.4)		
A/T	79 (20.5)		
T/T	4 (1.0)		
Variant allele frequency	11.3%		

**Table 2:** Main demographic and (co)morbidity characteristics of cases (experienced bleeding events) and controls (no bleeding events). Data are counts (percentages) or medians (quartiles, minimum-maximum).

	Cases (Bleeding)	Controls (No Bleeding)
N	71	314
*Demographics and renal function*		
Age (years)	74 (67–80; 30–88)	66 (57–72; 26–98)
Males	41 (57.8)	197 (62.7)
eGFR (mL/min/1.73 m^2^)	69 (47–85; 10–104)	76 (60–90; 15–125)
*Indication of rivaroxaban*		
Nonvalvular atrial fibrillation/atrial flutter	57 (80.3)	227 (72.3)
Peripheral artery disease	12 (16.9)	56 (17.8)
Venous thromboembolism	12 (16.9)	40 (12.7)
Ischemic stroke/TIA	7 (9.9)	50 (15.9)
Coronary heart disease/acute myocardial infarction	23 (32.4)	72 (22.9)
Thrombus in the left atrial apex/appendage	2 (2.8)	7 (2.2)
Adult congenital heart disease	0	6 (1.9)
Thrombophilia	2 (2.8)	6 (1.9)
*Daily rivaroxaban dose*		
1 × 20 mg	41 (57.8)	232 (73.9)
1 × 15 mg	20 (28.2)	33 (10.5)
1 × 10 mg	7 (9.9)	5 (1.6)
2 × 2.5 mg (total 5 mg)	3 (4.2)	44 (14.0)
*Comorbidities*		
Hypertension	64 (90.1)	253 (80.6)
Dyslipidemia	46 (64.8)	222 (70.7)
GI disease (peptic ulcer/diverticulosis/polyps/IBD)	32 (45.1)	24 (7.6)
Malignancy (solid organ/hematological)	20 (28.1) (16 + 4)	51 (16.3) (41 + 10)
Diabetes mellitus	14 (19.7)	71 (22.6)
Anxious or depressive disorder	9 (12.7)	30 (9.6)
Systemic inflammatory disease	4 (4.6)	7 (2.2)
Musculoskeletal pain syndromes	3 (4.2)	18 (5.7)

eGFR—estimated glomerular filtration rate; GI—gastrointestinal; IBD—inflammatory bowel disease; TIA—transient ischemic attack.

**Table 3:** Concomitant treatments in cases and controls. Data are count (percent).

	Cases (Bleeding)	Controls (No Bleeding)
N	71	314
Proton pump inhibitors	49 (69.0)	226 (72.0)
Platelet aggregation inhibitors	15 (21.1)	67 (21.1)
Dual antiplatelet therapy	5 (7.0)	10 (3.2)
CYP3A4 inhibitors		
0	47 (66.7)	186 (59.2)
At least 1 (1 to 3)	24 (33.8)	128 (40.8)
CYP3A4 substrates		
0	3 (4.2)	21 (6.7)
1–2	30 (42.3)	125 (39.8)
3 or more	38 (53.5)	168 (53.5)
CYP3A5 inhibitors		
0	64 (90.1)	269 (85.7)
At least 1 (1–2)	7 (9.9)	45 (14.3)
CYP3A5 substrates		
0	26 (36.6)	123 (39.2)
1–2	40 (56.3)	159 (50.6)
3 or more	5 (7.1)	32 (10.2)
CYP2J2 inhibitors		
0	55 (77.5)	241 (76.8)
1–2	16 (22.5)	73 (23.2)
ABCB1 inhibitors		
0	4 (5.6)	30 (9.5)
1–2	40 (56.3)	164 (52.3)
3 or more	27 (38.1)	120 (38.2)
ABCB1 inducers		
0	64 (90.1)	279 (88.8)
1–2	7 (9.9)	35 (11.2)
ABCB1 substrates		
0	4 (5.6)	24 (7.6)
1–2	43 (60.6)	182 (58.0)
3 or more	24 (33.7)	108 (34.4)
ABCG2 inhibitors		
0	24 (33.8)	92 (29.3)
1	47 (66.2)	222 (70.7)
ABCG2 substrates		
0	13 (18.3)	28 (8.9)
1–2	53 (74.6)	254 (80.9)
3 or more	5 (7.1)	32 (10.2)

**Table 4:** Prevalence of the investigated polymorphisms and genotype-predicted phenotypes in cases and controls. Data are count (percent).

	Cases (Bleeding)	Controls (No Bleeding)
N	71	314
CYP3A phenotype		
Rapid/intermediate metabolizer	68 (95.8)	294 (93.6)
Poor metabolizer	4 (4.2)	20 (6.4)
CYP2J2 phenotype (according to CYP2J*7)		
Normal function	66 (93.0)	273 (86.9)
Reduced function	5 (7.0)	41 (13.1)
*CYP2J2 A>T* (rs11572325)		
Wild type	60 (84.5)	242 (77.1)
Variant allele carrier	11 (15.5)	72 (22.9)
*ABCG2 c.421C>A*		
Wild type	55 (77.5)	259 (82.5)
Variant allele carrier	16 (22.5)	55 (17.5)
*ABCB1 c.1236C>T*		
Wild type	24 (33.8)	100 (31.8)
Variant allele carrier	47 (66.2)	214 (68.2)
*ABCB1 2677G>T/A*		
Wild type	28 (39.4)	129 (41.1)
Variant allele carrier	43 (60.6)	185 (58.9)
*ABCB1 3435C>T*		
Wild type	19 (26.8)	70 (22.3)
Variant allele carrier	52 (73.2)	244 (77.7)
*ABCB1 2482-2236G>A*		
Wild type	17 (23.9)	92 (29.3)
Variant allele carrier	54 (76.1)	222 (70.7)

**Table 5:** Raw and adjusted estimates of association (odds ratio, OR) between the investigated polymorphisms and the risk of bleeding.

Variant	Raw Data OR (95% CrI)	Balanced Data OR (95% CrI)
*CYP2J2* c.A>T variant allele vs. wild type	0.80 (0.50–1.38)	0.94 (0.55–1.65)
*ABCG2* c.421C>A variant allele vs. wild type	1.16 (0.70–1.88)	1.25 (0.76–2.12)
*ABCB1* c.1236C>T variant allele vs. wild type	1.12 (0.70–1.75)	1.05 (0.59–1.90)
*ABCB1* c.2677G>T/A variant allele vs. wild type	1.20 (0.76–1.95)	1.35 (0.81–2.07)
*ABCB1* c.3435C>T variant allele vs. wild type	1.04 (0.76–1.88)	0.85 (0.49–1.51)
*ABCB1* c.rs4148738 variant allele vs. wild type	1.20 (0.76–1.95)	1.12 (0.66–2.01)

Estimates for “raw” and “balanced” data were adjusted for age, eGFR, and gastrointestinal comorbidity.

**Table 6:** Comparison of key pharmacogenomic studies evaluating rivaroxaban clinical outcomes.

Study (Year)	N	Design	Main Variants	Key Finding	Consistency with Present Study
Wu et al. 2023 [12]	95	Prospective multicenter	*CYP3A4/5*, *ABCB1*, *ABCG2*	No bleeding association	Yes
Wang et al. 2025 [18]	SR	Systematic review	25 loci	Insufficient evidence for implementation	Yes
Li et al. 2024 [13]	165	Prospective	*CYP3A4/5*	*CYP3A4* variants associated with bleeding	Partial
Wang et al. 2025 [15]	228	Prospective PK research	*ABCB1* c.3435C>T, c.1236C>T, c.2677G>T/A	c.3435 T/T: higher VTE risk	No
Kim et al. 2023 [14]	293	Case-control	*ABCG2*, *ABCB1*	*ABCG2* rs3114018, *ABCB1* rs1045642 assoc. with bleeding	No
Campos-Staffico 2022 [19]	2364	Retrospective cohort	8 PK variants *ABCB1*, *ABCG2*, *CYP2J2*, *CYP3A4/5*	No PGx–DOAC bleeding association	Yes
Present study (2026)	385	Nested case-control	10 SNPs (5 genes)	No PGx signal; age + GI comorbidity dominate	—

PGx, pharmacogenomics; PK, pharmacokinetics; SR, systematic review; VTE, venous thromboembolism.

## Data Availability

The original contributions presented in this study are included in the article/Supplementary Material. Further inquiries can be directed to the corresponding author.

## Supplementary Materials

The following supporting information can be downloaded at: https://www.mdpi.com/article/10.3390/pharmaceutics18070884/s1, Figure S1. Distribution of time on rivaroxaban for cases (experienced bleeding) and controls (no bleeding). Table S1. Major characteristics of cases (bleeding) and controls (no bleeding) after covariate balancing for the purpose of assessment of the relationship between polymoprhism *CYP2J2A>T* and bleeding. Data are weighted percentages. Standardized mean differences (d) < 0.1 indicates adequate balance. Table S2. Major characteristics of cases (bleeding) and controls (no bleeding) after covariate balancing for the purpose of assessment of the relationship between polymoprhism *ABCG2 421C>A* and bleeding. Data are weighted percentages. Standardized mean differences (d) < 0.1 indicates adequate balance. Table S3. Major characteristics of cases (bleeding) and controls (no bleeding) after covariate balancing for the purpose of assessment of the relationship between polymoprhism *ABCB1 1236C>T* and bleeding. Data are weighted percentages. Standardized mean differences (d) < 0.1 indicates adequate balance. Table S4. Major characteristics of cases (bleeding) and controls (no bleeding) after covariate balancing for the purpose of assessment of the relationship between polymoprhism *ABCB1 2677G>T/A* and bleeding. Data are weighted percentages. Standardized mean differences (d) < 0.1 indicates adequate balance. Table S5. Major characteristics of cases (bleeding) and controls (no bleeding) after covariate balancing for the purpose of assessment of the relationship between polymoprhism *ABCB1 3435C>T* and bleeding. Data are weighted percentages. Standardized mean differences (d) < 0.1 indicates adequate balance. Table S6. Major characteristics of cases (bleeding) and controls (no bleeding) after covariate balancing for the purpose of assessment of the relationship between polymoprhism *ABCB1 2482-2236G>A* and bleeding. Data are weighted percentages. Standardized mean differences (d) < 0.1 indicates adequate balance.
