# Supplementary material for: Pharmacogenomics of Rivaroxaban: Association of CYP3A4, CYP3A5, CYP2J2, ABCB1, and ABCG2 Variants with Bleeding and Thrombotic Outcomes in Real-World Clinical Practice
Source: Pharmaceutics. 2026 Jul 20;18(7):884. doi: 10.3390/pharmaceutics18070884 (PMC13416040; doi:10.3390/pharmaceutics18070884)
Supplement: Supplementary file 1 [file pharmaceutics-18-00884-s001.zip › pharmaceutics-4391916-supplementary.pdf]

Supplemental Material

Slišković et al. Pharmacogenomics of Rivaroxaban: Association of *CYP3A4, CYP3A5, CYP2J2, ABCB1*, and *ABCG2* Variants with Bleeding and Thrombotic Outcomes in Real-World Clinical

Figure S1. Distribution of time on rivaroxaban in patients experiencing bleeding (cases) and those without bleeding events (controls).

Table S1. Major characteristics of cases (bleeding) and controls (no bleeding) after covariate balancing for the purpose of assessment of the relationship between polymoprhism *CYP2J2A>T* and bleeding.

Table S2. Major characteristics of cases (bleeding) and controls (no bleeding) after covariate balancing for the purpose of assessment of the relationship between polymoprhism *ABCG2 421C>A* and bleeding.

Table S3. Major characteristics of cases (bleeding) and controls (no bleeding) after covariate balancing for the purpose of assessment of the relationship between polymoprhism *ABCB1 1236C>T* and bleeding.

Table S4. Major characteristics of cases (bleeding) and controls (no bleeding) after covariate balancing for the purpose of assessment of the relationship between polymoprhism *ABCB1 2677G>T/A* and bleeding.

Table S5. Major characteristics of cases (bleeding) and controls (no bleeding) after covariate balancing for the purpose of assessment of the relationship between polymoprhism *ABCB1 3435C>T* and bleeding. Data are weighted percentages. Standardized mean differences (d) <0.1 indicates adequate balance.

Table S6. Major characteristics of cases (bleeding) and controls (no bleeding) after covariate balancing for the purpose of assessment of the relationship between polymoprhism *ABCB1 2482-2236G>A* and bleeding.

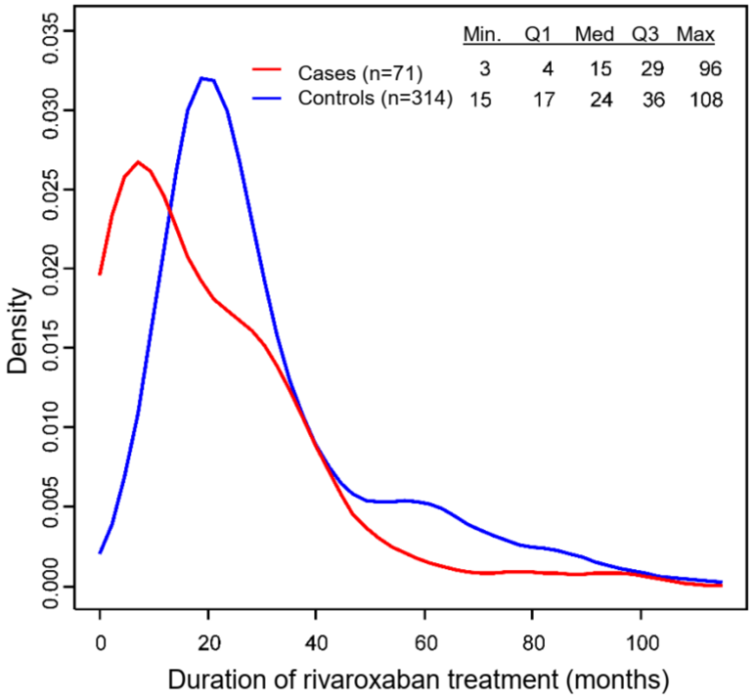

Figure S1. Distribution of time on rivaroxaban for cases (experienced bleeding) and controls (no bleeding).

Table S1. Major characteristics of cases (bleeding) and controls (no bleeding) after covariate balancing for the purpose of assessment of the relationship between polymoprhism *CYP2J2A>T* and bleeding. Data are weighted percentages. Standardized mean differences (d) <0.1 indicates adequate balance.

	Cases[1]	Controls[2]	d
N	71	314	---
Variables used for balancing			
Males	61.4	61.8	-0.008
Females	38.6	38.2	0.008
Rivaroxaban dose 1x15 or 1x20 mg/day	85.0	84.7	0.008
Rivaroxaban dose 1x10 or 2x2.5 mg/day	15.0	15.3	-0.008
Proton pump inhibitors	70.3	71.4	-0.024
Platelet inhibitors	21.8	21.3	0.012
Atrial fibrillation	77.2	73.8	0.081
Hypertension	85.2	82.3	0.081
Dyslipidaemia	70.5	69.6	0.018
Diabetes mellitus	22.9	22.1	0.021
History of malignancy	20.6	17.7	0.072
CYP3A poor metabolizer	4.7	6.0	-0.059
CYP3A intermediate-rapid metabolizer	95.3	94.0	0.059
CYP2J2 reduced function according to *CYP2J2*7*	9.9	12.0	-0.067
CYP2J2 normal function according to *CYP2J2*7*	90.1	88.0	0.067
ABCG2 421C>A variant allele	18.9	18.4	0.010
ABCG2 421C>A wild type	81.1	81.6	-0.010
ABCB1 1236C>T variant allele	67.4	67.8	-0.008
ABCB1 1236C>T wild type	32.6	32.2	0.008
ABCB1 2677G>T/A variant allele	58.4	59.2	-0.017
ABCB1 2677G>T/A wild type	41.6	40.8	0.017
ABCB1 3435C>T variant allele	75.1	76.9	-0.040
ABCB1 3435C>T wild type	24.9	23.1	0.040
ABCB1 2482-2236G>A variant allele	72.6	71.7	0.020
ABCB1 2482-2236G>A wild type	27.4	28.3	-0.020
Polymorphism of interest			
CYP2J2A>T variant allele	20.2	21.7	---

[1]Weights: mean=1, mean absolute deviation 0.411, range 0.346-1.468; Entropy 0.110; Effective sample size=58.8

[2]Weights: mean=1, mean absolute deviation 0.096, range 0.600-1.424; Entropy 0.008; Effective sample size=309.0

Tablica S2. Major characteristics of cases (bleeding) and controls (no bleeding) after covariate balancing for the purpose of assessment of the relationship between polymoprhism *ABCG2 421C>A* and bleeding. Data are weighted percentages. Standardized mean differences (d) <0.1 indicates adequate balance.

	Cases[1]	Controls[2]	d
N	71	314	---
Variables used for balancing			
Males	60.6	61.8	-0.024
Females	39.4	38.2	0.024
Rivaroxaban dose 1x15 or 1x20 mg/day	85.1	84.7	0.012
Rivaroxaban dose 1x10 or 2x2.5 mg/day	14.9	15.3	-0.012
Proton pump inhibitors	70.7	71.4	-0.017
Platelet inhibitors	20.9	21.3	-0.008
Atrial fibrillation	76.7	73.8	0.071
Hypertension	84.8	82.3	0.072
Dyslipidaemia	69.7	69.6	0.002
Diabetes mellitus	22.8	22.1	0.017
History of malignancy	19.9	17.7	0.056
CYP3A poor metabolizer	4.9	6.0	-0.048
CYP3A intermediate-rapid metabolizer	95.1	94.0	0.048
CYP2J2 reduced function according to *CYP2J2*7*	10.4	12.0	-0.050
CYP2J2 normal function according to *CYP2J2*7*	89.6	88.0	0.050
CYP2J2A>T variant allele	20.6	21.6	-0.025
CYP2J2A>T wild type	79.4	78.4	0.025
ABCB1 1236C>T variant allele	68.2	67.8	0.007
ABCB1 1236C>T wild type	31.8	32.2	-0.007
ABCB1 2677G>T/A variant allele	60.0	59.2	0.016
ABCB1 2677G>T/A wild type	40.0	40.8	-0.016
ABCB1 3435C>T variant allele	76.0	76.9	-0.019
ABCB1 3435C>T wild type	24.0	23.1	0.019
ABCB1 2482-2236G>A variant allele	73.2	71.7	0.034
ABCB1 2482-2236G>A wild type	26.8	28.3	-0.034
Polymorphism of interest			
ABCG2 421C>A variant allele	24.3	17.5	---

[1]Weights: mean=1, mean absolute deviation 0.387, range 0.339-1.565; Entropy 0.104; Effective sample size=59.3

[2]Weights: mean=1, mean absolute deviation 0.094, range 0.604-1.350; Entropy 0.008; Effective sample size=309.2

Table S3. Major characteristics of cases (bleeding) and controls (no bleeding) after covariate balancing for the purpose of assessment of the relationship between polymoprhism *ABCB1 1236C>T* and bleeding. Data are weighted percentages. Standardized mean differences (d) <0.1 indicates adequate balance.

	Cases[1]	Controls[2]	d
N	71	314	---
Variables used for balancing			
Males	61.5	61.8	-0.006
Females	38.5	38.2	0.006
Rivaroxaban dose 1x15 or 1x20 mg/day	85.1	84.7	0.011
Rivaroxaban dose 1x10 or 2x2.5 mg/day	14.9	15.3	-0.011
Proton pump inhibitors	70.2	71.4	-0.026
Platelet inhibitors	21.5	21.3	0.006
Atrial fibrillation	77.0	73.8	0.076
Hypertension	85.1	82.3	0.080
Dyslipidaemia	70.1	69.6	0.011
Diabetes mellitus	22.7	22.1	0.016
History of malignancy	20.3	17.7	0.065
CYP3A poor metabolizer	4.7	6.0	-0.056
CYP3A intermediate-rapid metabolizer	95.3	94.0	0.056
CYP2J2 reduced function according to *CYP2J2*7*	10.1	12.0	-0.062
CYP2J2 normal function according to *CYP2J2*7*	89.9	88.0	0.062
CYP2J2A>T variant allele	19.9	21.6	-0.043
CYP2J2A>T wild type	80.1	78.4	0.043
ABCG2 421C>A variant allele	19.1	18.4	0.015
ABCG2 421C>A wild type	80.9	81.6	-0.015
ABCB1 2677G>T/A variant allele	58.3	59.2	-0.019
ABCB1 2677G>T/A wild type	41.7	40.8	0.019
ABCB1 3435C>T variant allele	75.4	76.9	-0.034
ABCB1 3435C>T wild type	24.6	23.1	0.034
ABCB1 2482-2236G>A variant allele	72.2	71.7	0.012
ABCB1 2482-2236G>A wild type	27.8	28.3	-0.012
Polymorphism of interest			
ABCB1 1236C>T variant allele	68.1	68.9	---

[1]Weights: mean=1, mean absolute deviation 0.426, range 0.355-1.543; Entropy 0.117; Effective sample size=58.1

[2]Weights: mean=1, mean absolute deviation 0.091, range 0.670-1.426; Entropy 0.007; Effective sample size=309.6

Table S4. Major characteristics of cases (bleeding) and controls (no bleeding) after covariate balancing for the purpose of assessment of the relationship between polymoprhism *ABCB1 2677G>T/A* and bleeding. Data are weighted percentages. Standardized mean differences (d) <0.1 indicates adequate balance.

	Cases[1]	Controls[2]	d
N	71	314	---
Variables used for balancing			
Males	60.9	61.8	-0.018
Females	39.1	38.2	0.018
Rivaroxaban dose 1x15 or 1x20 mg/day	85.2	84.7	0.016
Rivaroxaban dose 1x10 or 2x2.5 mg/day	14.8	15.3	-0.016
Proton pump inhibitors	70.5	71.4	-0.021
Platelet inhibitors	21.3	21.3	0.000
Atrial fibrillation	77.1	73.8	0.078
Hypertension	85.0	82.3	0.077
Dyslipidaemia	70.2	69.6	0.013
Diabetes mellitus	22.8	22.1	0.017
History of malignancy	19.5	17.7	0.045
CYP3A poor metabolizer	4.9	6.0	-0.048
CYP3A intermediate-rapid metabolizer	95.1	94.0	0.048
CYP2J2 reduced function according to *CYP2J2*7*	11.1	12.0	-0.026
CYP2J2 normal function according to *CYP2J2*7*	88.9	88.0	0.026
CYP2J2A>T variant allele	21.2	21.6	-0.009
CYP2J2A>T wild type	78.8	78.4	0.009
ABCG2 421C>A variant allele	19.4	18.4	0.024
ABCG2 421C>A wild type	80.6	81.6	-0.024
ABCB1 1236C>T variant allele	66.6	67.8	-0.025
ABCB1 1236C>T wild type	33.4	32.2	0,025
ABCB1 3435C>T variant allele	75.3	76.9	-0.037
ABCB1 3435C>T wild type	24.7	23.1	0.037
ABCB1 2482-2236G>A variant allele	71.5	71.7	-0.003
ABCB1 2482-2236G>A wild type	28.5	28.3	0.003
Polymorphism of interest			
ABCB1 2677G>T/A variant allele	64.5	59.2	---

[1]Weights: mean=1, mean absolute deviation 0.426, range 0.363-1.623; Entropy 0.118; Effective sample size=57.8

[2]Weights: mean=1, mean absolute deviation 0.097, range 0.603-1.424; Entropy 0.008; Effective sample size=309.0

Table S5. Major characteristics of cases (bleeding) and controls (no bleeding) after covariate balancing for the purpose of assessment of the relationship between polymoprhism *ABCB1 3435C>T* and bleeding. Data are weighted percentages. Standardized mean differences (d) <0.1 indicates adequate balance.

	Cases[1]	Controls[2]	d
N	71	314	---
Variables used for balancing			
Males	62.0	61.8	-0.004
Females	38.0	38.2	0.004
Rivaroxaban dose 1x15 or 1x20 mg/day	85.0	84.7	0.019
Rivaroxaban dose 1x10 or 2x2.5 mg/day	15.0	15.3	-0.019
Proton pump inhibitors	70.0	71.4	-0.026
Platelet inhibitors	23.5	20.7	0.002
Atrial fibrillation	75.9	73.8	0.057
Hypertension	84.8	82.3	0.075
Dyslipidaemia	70.6	69.6	0.015
Diabetes mellitus	22.9	22.1	0.018
History of malignancy	19.1	17.7	0.045
CYP3A poor metabolizer	5.1	6.0	-0.036
CYP3A intermediate-rapid metabolizer	94.9	94.0	0.036
CYP2J2 reduced function according to *CYP2J2*7*	10.9	12.0	-0.041
CYP2J2 normal function according to *CYP2J2*7*	89.1	88.0	0.041
CYP2J2A>T variant allele	20.8	21.6	-0.027
CYP2J2A>T wild type	79.2	78.4	0.027
ABCG2 421C>A variant allele	18.5	18.4	0.008
ABCG2 421C>A wild type	81,5	81.6	-0.008
ABCB1 1236C>T variant allele	67.5	67.8	-0.012
ABCB1 1236C>T wild type	32.5	32.2	0.012
ABCB1 2677G>T/A variant allele	58.9	59.2	-0.012
ABCB1 2677G>T/A wild type	41.1	40.8	0.012
ABCB1 2482-2236G>A variant allele	72.2	71.7	0.005
ABCB1 2482-2236G>A wild type	27.8	28.3	-0.005
Polymorphism of interest			
ABCB1 3435C>T variant allele	71.4	77.9	---

[1]Weights: mean=1, mean absolute deviation 0.393, range 0.383-1.644; Entropy 0.104; Effective sample size=59.1

[2]Weights: mean=1, mean absolute deviation 0.095, range 0.638-1.417; Entropy 0.007; Effective sample size=309.4

Table S6. Major characteristics of cases (bleeding) and controls (no bleeding) after covariate balancing for the purpose of assessment of the relationship between polymoprhism *ABCB1 2482-2236G>A* and bleeding. Data are weighted percentages. Standardized mean differences (d) <0.1 indicates adequate balance.

	Cases[1]	Controls[2]	d
N	71	314	---
Variables used for balancing			
Males	60.9	61.8	-0.018
Females	39.1	38.2	0.018
Rivaroxaban dose 1x15 or 1x20 mg/day	85.2	84.7	0.016
Rivaroxaban dose 1x10 or 2x2.5 mg/day	14.8	15.3	-0.016
Proton pump inhibitors	70.9	71.4	-0.012
Platelet inhibitors	21.8	21.3	0.012
Atrial fibrillation	77.2	73.8	0.081
Hypertension	85.1	82.3	0.079
Dyslipidaemia	70.6	69.6	0.021
Diabetes mellitus	23.1	22.1	0.024
History of malignancy	20.4	17.7	0.068
CYP3A poor metabolizer	4.8	6.0	-0.052
CYP3A intermediate-rapid metabolizer	95.2	94.0	0.052
CYP2J2 reduced function according to *CYP2J2*7*	10.0	12.0	-0.064
CYP2J2 normal function according to *CYP2J2*7*	90.0	88.0	0.064
CYP2J2A>T variant allele	19.9	21.6	-0.043
CYP2J2A>T wild type	80.1	78.4	0.043
ABCG2 421C>A variant allele	19.2	18.4	0.017
ABCG2 421C>A wild type	80.8	81.6	-0.017
ABCB1 1236C>T variant allele	66.9	67.8	-0.018
ABCB1 1236C>T wild type	33.1	32.2	0.018
ABCB1 2677G>T/A variant allele	58.0	59.2	-0.025
ABCB1 2677G>T/A wild type	42.0	40.8	0.025
ABCB1 3435C>T variant allele	75.1	76.9	-0.042
ABCB1 3435C>T wild type	24.9	23.1	0.042
Polymorphism of interest			
ABCB1 2482-2236G>A variant allele	74.6	70.2	---

[1]Weights: mean=1, mean absolute deviation 0.414, range 0.349-1.549; Entropy 0.112; Effective sample size=58.5

[2]Weights: mean=1, mean absolute deviation 0.086, range 0.628-1.406; Entropy 0.006; Effective sample size=310.2
